# Evaluating physical activities in clinical diabetes: lifestyle scores hypothesis

**DOI:** 10.1017/S1463423624000434

**Published:** 2024-10-17

**Authors:** Phillip Bwititi, Solomon Egwuenu, Echinei Oshionwu, John Okuzor, Alex Odufu, Charles Ofili, Ezekiel Uba Nwose

**Affiliations:** 1 School of Dentistry & Medical Sciences, Charles Sturt University, New South Wales, Australia; 2 College of Medicine & Health Sciences, Novena University Ogume, Ogume, Nigeria; 3 Global Medical Research & Development Organization (GMRDO) Group, Abbi Delta State, Nigeria; 4 School of Health & Medical Sciences, University of Southern Queensland, Toowoomba Australia

**Keywords:** investigative techniques, preventive medicine in metabolic syndrome, primary healthcare

## Abstract

**Background:**

The concept of lifestyle-based risk scores is known but not evaluated in most rural communities of low- to mid-income countries. This study investigated the correlation of lifestyle scores with health indices.

**Methods:**

This was a descriptive cross-sectional investigation. A total of 203 participants (141 females and 62 males), 18–90 years, had anthropometric assessments and lifestyle scores determined from a 12-item framework. Data analysis included average age in different health conditions, lifestyle scores in age groups, and correlations with age.

**Results:**

Average age of healthy subpopulation was 39 years while diabetes, hypertension, and obesity subpopulations were 58, 64, and 56 years, respectively. The percentage of participants whose activities of daily living (ADL) were unaffected by ill-health decreased with age (*P* < 0.0001), and lifestyle scores also decreased with age (*P* < 0.01) and negatively correlated with physical activities.

**Conclusion:**

This report contributes to diabetes cardiovascular complications management. Sedentary ADL factors need integration in healthy lifestyle education especially among the elderly.

## Introduction

Lifestyle including physical activities is integral to diabetes self-management (Booth *et al.*, 2013). Several clinical trials have investigated the practicability and efficacy of lifestyle interventions in preventing diabetes in people with prediabetes (Buijsse *et al.*, 2011; Papandonatos *et al.*, 2015; Perreault *et al.*, 2012). Such reports demonstrate that positive lifestyle modifications are more effective in reducing the incidence of diabetes development by comparison with standard treatment (Kerrison *et al.*, 2017; Knowler *et al.*, 2009; Perry *et al.*, 2023), although some patients may remain prediabetes (DeFronzo & Abdul-Ghani, 2011; Perreault *et al.*, 2012). Less discussed is the reason for failure of lifestyle intervention among the participants who remain prediabetic and/or progress to diabetes. The Diabetes Prevention Program (DPP) and the Diabetes Prevention Program Outcomes Study (DPPOS) reported that intensive lifestyle intervention was more effective than management using drugs and better in the elderly (Knowler *et al.*, 2009). Therefore, an addendum was added to the research to ascertain compliance of vigorous lifestyle intervention in younger people so that weight loss is sustained over a prolonged duration (Misra, 2009). It is important to evaluate older adults, regarding the potential correlation *vis-à-vis* the impact of lifestyle changes attributable to old age, since this group is vulnerable to diabetes and its cardiovascular complication.

Further, obesity, among others, is caused by a combination of decreased energy expenditure and increased energy intake. It is acknowledged that the propensity for obesity to cause diabetes is exacerbated by physical inactivity (Faghri *et al.*, 2015; Martinez-Gonzalez *et al.*, 1999). Reports highlight that exercise therapy may be more effective in younger people (Cartee, 1994; Johnson *et al.*, 2014), whereas intensive lifestyle intervention shows better results among older adults (Knowler *et al.*, 2009; Misra, 2009). Thus, we propound a *lifestyle scores’ hypothesis* to evaluate how changes in overall lifestyle scores relate with health encompassing diabetes control and cardiovascular disease indices. The significance of expected outcome is in the investigative techniques and preventive medicine for individuals living with metabolic syndrome and at risk of cardiovascular complications.

## International overview of the theme

Healthy dietary habit hypothesis dates from the 1940s and evolved to encompass the United States Department of Agriculture (USDA) food pyramid recommendation, changes in the consideration of fat content and size of foods, and the primary prevention guidelines (Fischer *et al.*, 2020). Several trials (Fleming & Godwin, 2008; Hesselink *et al.*, 2013; Pape *et al.*, 2022; Tokunaga-Nakawatase *et al.*, 2014; Yang *et al.*, 2014) have contributed to progress the notion of lifestyle modification in diabetes self-management. On the discourse of lifestyle score, it has been determined that a lifestyle-based model can be considered in assessing diabetes risk (Buss *et al.*, 2021). It is pertinent to note that there is an Australian model of risk assessment for type 2 diabetes mellitus amongst other tools (Buijsse *et al.*, 2011; Chen *et al.*, 2010), and lifestyle predictors are easy for patients to understand (Buss *et al.*, 2021).

It is recommended that ‘discriminatory performance is more heterogeneous and generally weaker in external populations, which suggests that risk scores may need to be validated within the population in which they are intended to be used’ (Buijsse *et al.*, 2011). This therefore calls for health facilities and community health services involved in diabetes care to develop their own population-based scores. Further, changes in overall lifestyle scores relate to health conditions such as metabolic syndrome (Martin *et al.*, 2016; Melaku *et al.*, 2023), but evaluation of how these changes occur in specific populations such as Nigeria requires investigation.

The objective of this work is to evaluate how lifestyle scores change with components of metabolic syndrome, especially type 2 diabetes mellitus. This includes an assessment of the correlation between lifestyle score and physical activities.

## Methods

### Design and setting

This was a cross-sectional quantitative study involving recruitment by opportunistic sampling. The study setting was the community health outreach of diabetes screening at a Catholic hospital in Abbi Delta State, Nigeria. Participants were recruited from individuals who attended the diabetes screening clinic.

### Data collection

Data were collected during the community health outreach in December 2016 and January 2017. A structured questionnaire collected information as part of an ongoing project. The questionnaire had various sections: A—Background of participant, B—General health status including previous diabetes and/or CVD, and C—Symptoms. Lifestyle variables including daily and physical activities made up sections D and E, while section F collected data on dietary habits.

This study was more focused on the variables of daily and physical activities; hence, sections A, B, D, and E constituted the data sources. Obesity was determined from weight and height. The 12-item questions on lifestyle activities are relatively different from the common health scorecard (Ratzan *et al.*, 2013) and were adopted from standard health and wellbeing questionnaires (Hooker, 2013; World Health Organization, 2017).

### Determination of ‘lifestyle scores’

Method is according to the protocol of *Evaluation of inter-current illness intervening lifestyle in stratified age groups* (Nwose *et al.*, 2018). Therefore, for distinction between the variables in daily versus physical activities, daily activities comprised occupational ADL events including going to work, performing social activities, hobbies, house chores, and errands/shopping. Alcohol and cigarette smoking were included as ‘negative effect’ lifestyle daily activities. The physical activities were purposive exercises including bicycling, stretching, swimming, walking, and any other form.

### Statistical analysis of data

This study included 203 participants (comprised of 141 females and 62 males), as described in results (Table 1). Data generated were mainly quantitative variables and analyzed using Microsoft Excel Data Analysis ToolPak 2010. Besides descriptive statistics, other statistical analyses included ANOVA of lifestyle scores in age groups, Student’s *t* test analysis of lifestyle scores between ‘healthy versus DM’ groups, and Pearson’s correlation coefficient between age and lifestyle scores.

**Table 1.** Summary of metabolic syndrome components and ‘no’ responses in age groups (Nwose *et al.*, 2018)

Age range (years)	18–39	40–59	60–69	70–79	80+
*N*	40	53	46	35	29
Hypertension^*^	1	12	14	12	9
Diabetes^*^	1	4	3	1	1
Dyslipidaemia^*^	0	1	0	0	0
Obesity (BMI > 30)	3	8	5	1	2
Daily activity 1	26	33	16	10	9
Daily activity 2	28	32	22	17	9
Daily activity 3	29	32	17	14	8
Daily activity 4	30	35	17	12	6
Daily activity 5	33	35	19	13	8
Daily activity 6 (smoking)	37	47	43	35	27
Daily activity 7 (alcohol)	32	34	40	24	23
Physical activity 1	28	33	31	28	24
Physical activity 2	4	3	5	2	9
Physical activity 3	31	49	42	33	29
Physical activity 4	17	30	23	15	20
Physical activity 5	39	50	46	35	29

* Based on being clinically diagnosed as reported by client.

### Bias

The same questions on lifestyle were applied to all participants regardless of age. Thus, the ‘lifestyle score’ for each participant was without bias.

## Results

**Figure 1.**
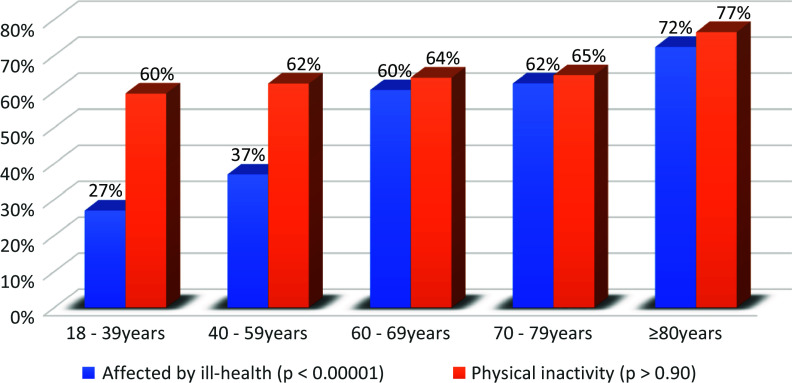
Summary of ill-health and physical inactivity in each age group.

Descriptive statistics showing percentage of [no] responses to questions on daily and physical activities, as well as prevalence of metabolic syndrome in age groups, have been reported (Nwose *et al.*, 2018). The absolute numbers of metabolic syndrome (chronic disease) components in the stratified age groups, as well as the absolute numbers of respondents who answered [no] to the questions on daily and physical activities (Table 1), and the percentage of ill-health and physical inactivity in each group are shown (Figure 1).

**Figure 2.**
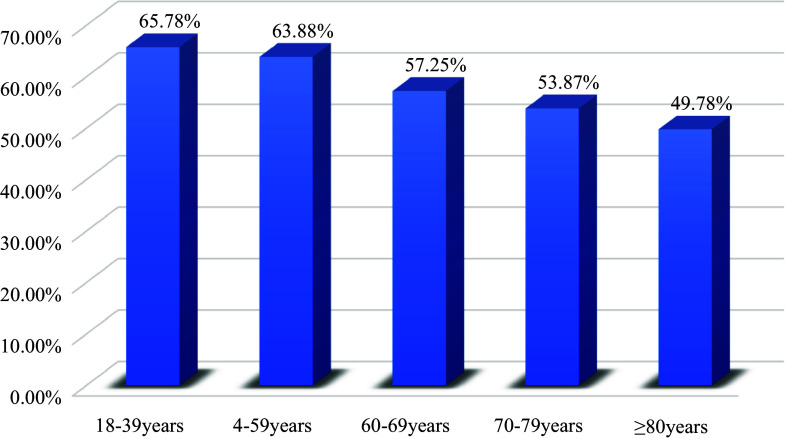
Percentage average of ‘lifestyle scores’ in age groups (*P* < 0.01).

The percentage of the age groups that constituted disease subpopulations was significantly different (*P* < 0.0001), but the distribution of age groups into health conditions showed no statistical difference. The average ‘lifestyle scores’ in different age groups were significantly different (Figure 2; *P* < 0.01). Comparison of average points from all 12 activities’ questions between different age groups showed no statistical difference in physical activities, except when limited to D1 – D5 variables.

**Figure 3.**
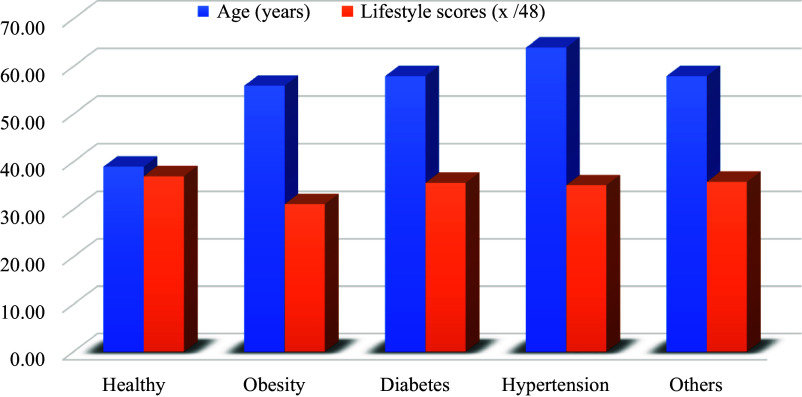
Averages of age and ‘lifestyle scores’ compared between health conditions (*P* > 0.50).

The *t* test analysis of differences in responses between ‘healthy vs. DM’ groups showed no statistical significance (*P* > 0.90) and no statistical difference in lifestyle scores among health conditions. The average age of the healthy subpopulation was 39 years, while for diabetes and hypertension, they were 58 years and 64 years, respectively (Figure 3).

On correlations, the results showed that physical activity variables were negatively correlated with age, but only E2 (walking as a form of exercise) was moderately important. Influences of ill-health on daily activities were moderately and positively correlated with age (Table 2).

**Table 2.** Correlation analysis

	*Age*	*D1*	*D2*	*D3*	*D4*	*D5*	*D6*	*D7*	*E1*	*E2*	*E3*	*E4*	*E5*
Age	1												
D1	0.3625	1.0000											
D2	0.2444	0.5942	1										
D3	0.3163	0.6489	0.7601	1.0000									
D4	0.3342	0.6580	0.6131	0.7153	1								
D5	0.3106	0.6199	0.6513	0.7079	0.8035	1.0000							
D6	–0.0652	–0.1284	0.0640	–0.0217	–0.0328	–0.0159	1						
D7	–0.0140	–0.1612	–0.0565	–0.0732	–0.0576	–0.0815	0.5037	1.0000					
E1	–0.0799	–0.0247	0.0328	–0.0029	–0.0694	–0.0757	–0.0219	–0.0006	1				
E2	–0.2537	–0.3129	–0.1725	–0.2337	–0.2650	–0.1589	0.1017	0.1701	0.3299	1.0000			
E3	–0.1798	–0.1295	–0.0032	–0.0588	–0.0548	–0.0136	0.2994	0.2025	–0.0352	0.2147	1		
E4	–0.0355	–0.0364	0.0361	0.0338	–0.0639	0.0005	0.0204	–0.0107	0.2150	0.2576	–0.0164	1.0000	
E5	–0.0807	–0.1135	–0.0985	–0.1028	–0.1032	–0.0950	0.3367	0.2543	0.0401	0.0845	0.3307	–0.0502	1
LS	–0.1905	–0.1294	–0.0528	–0.0835	–0.1697	–0.0775	–0.2168	–0.2657	0.5776	0.6593	0.2465	0.6627	0.0508

*Note*: LS = lifestyle score; D = daily activities; E = physical activities.

## Discussion

The results show that the highest percentage of group 1 (18–39 years) had daily work activities unaffected by ill-health, relative to the lowest percentages in groups 4 and 5 (70–79 years) and over 80 years, respectively (Figure 1). The average ‘lifestyle scores’ in different age groups were statistically different (Figure 2), and this can be interpreted that lifestyle scores decrease with age. There was no difference in lifestyle scores between the different health subgroups (Fig 3), and relative to the observations presented in Figure 2, further, there was no statistical difference in lifestyle scores between health conditions. Such observations imply the need of patient’s age in the definition of healthy lifestyle. It has been suggested that the person-centred approach should be used in counselling patients about physical activities (Sanghamitra *et al.*, 2019). What this study contributes is the concept of lifestyle scores, which is albeit a known idea yet to be fully applied in diabetes self-management.

The average age of the healthy subpopulation was 39 years, while for diabetes and hypertension, the averages were 58 years and 64 years, respectively. Studies have demonstrated that taking part in social activity is significantly linked to general body health and that such association is independent of socioeconomic status or comorbidities (Haeuchi *et al.*, 2016). This Nigerian community-dwelling population report contributes that aging positively correlates with impaired daily routine activities and is negatively associated with physical activities (Table 2).

Previous observations in the studied population were that despite smoking practice being low, it still possibly accounted for high risk for elevated blood levels of glucose as well as triglycerides in smokers (Nwose *et al.*, 2018). Further, the widespread alcohol intake coupled with unhealthy food choices may contribute to the high prevalence of indices of cardiovascular disease, such as diabetes mellitus. Health education should highlight the benefits of consuming fruits and vegetables and lowering intake of processed oils and the negative effects of active and passive smoking.

## Limitation

As indicated earlier, ‘the same questions on lifestyle were applied to all participants regardless of age. Thus, the “lifestyle score” for each participant was without bias’. Although the factor of age has been analyzed, it is acknowledged that there are limitations due to age, which have not been evaluated.

### Relevance to primary healthcare and implications for policy and practice

#### Importance for health system’s policy

Recognizing the barriers and facilitators of lifestyle in diabetes self-management can help refine diabetes education programmes (Booth *et al.*, 2013). The World Health Organization (WHO) noted harmful foods, lack of exercise and other forms of physical activities, tobacco smoking/consumption, and high alcohol intake as risk factors for CVD. The WHO report on global targets for 2025 to decrease the prevalence of harmful behaviours that increase non-communicable diseases (NCDs) highlights that countries speed up the implementation of preventive measures. However, in Nigeria, there appears to be no working health policy or plan to reduce smoking and unhealthy food consumption and/or to promote beneficial foods and exercise as well as other daily activities (World Health Organization, 2014). Further, few epidemiological studies on patterns of unhealthy lifestyles in Nigeria have been done. Understanding the patterns of distribution of diet and lifestyle habits and their interplay with other cardiovascular indices can provide empirical evidence to plan intervention strategies and engage policymakers.

#### Importance for primary healthcare professional practice

The observation from a survey on adherence to exercise prescription indicates health as a major limiting factor (Noon *et al.*, 2018). It is also reported that the elderly and vulnerable adults with relatively healthier lifestyle habits are more likely to benefit from primary preventative care interventions (Raymond *et al.*, 2012). These observations are practice implications in the discourse of lifestyle with regard to risk management. This study adds that aging, as a non-modifiable physiological factor, interferes with the capacity to undertake routine physical ADL, which then causes lifestyle changes. This is important for reflective primary health practice, especially in psychological counselling support for the elderly individuals attending community health services.

#### Importance for research and development

Lifestyle intervention regimen should consider age in addition to focusing on abilities or adherence to maintain daily routines especially for investigative techniques and preventive medicine in cardiovascular complications among individuals living with metabolic syndrome (Gómez-Martínez *et al.*, 2022; Martinez-Gonzalez *et al.*, 1999; Perry *et al.*, 2023). This is an advancement in the concept of lifestyle scores idea and recent report highlights (Melaku *et al.*, 2023). Thus, this is a novel application of a known idea that lifestyle scores could be a useful investigative technique for evaluation of clients in primary healthcare settings.

#### Importance for society

It is accepted that the personality of an individual living with diabetes is impacted by an unhealthy lifestyle (Mommersteeg *et al.*, 2010). Therefore, beyond risk assessment, a group or society of individuals high in prevalence of metabolic syndrome would benefit from community health services that employ lifestyle scores as part of their management tool. This opinion is supported by a pilot study from India (West-Pollak *et al.*, 2014), which showed that an intervention programme on lifestyle based on education improves diabetes control.

#### Importance for target individuals (the patients)

It is reported that lifestyle predictors are easier for patients to understand (Buss *et al.*, 2021). Hence, an immediate impact of this study to patients of the primary healthcare is in patient education. That is, patients can benefit from the adoption of this during the provision of health education in preventive medicine practice at primary healthcare. Another potential benefit for individuals is motivation for lifestyle modification (Ross & VanNortwick, 2022), that is, knowing how and which dietary and/or ADL changes have improved their lifestyle score, thus mitigating the health risk.

## Conclusion

This report contributes to data in view of the ongoing discourse that impaired capacity for routine daily activities increases with aging. Studies on lifestyle intervention have consistently reported effectiveness albeit with less discussion on the behavioural change wheel *vis-à-vis* capacity, motivation, and opportunity. This study recommends that a strategy/plan to decrease physical inactivity and/or promote physical activity needs to consider the limitations due to aging.
